# Diet and physical activity behavior among users of prescription weight loss medications

**DOI:** 10.1186/1479-5868-1-17

**Published:** 2004-12-23

**Authors:** Heidi Michels Blanck, Laura Kettel Khan, Mary K Serdula

**Affiliations:** 1 Division of Nutrition & Physical Activity, Centers for Disease Control and Prevention, Atlanta, GA, USA

## Abstract

**Background:**

There is limited population-based data on diet and physical activity behaviors and weight loss among users of prescription weight loss medications. Most findings are from clinical settings or from research that includes organized behavioral programs.

**Methods:**

We analyzed data from the 1998 Behavioral Risk Factor Surveillance System, an annual telephone survey conducted in all fifty states, the District of Columbia and Puerto Rico. The sample consisted of 135,435 noninstitutionalized adults aged 18 years old and older. We determined the prevalence and odds of prescription weight loss medication use, odds of 10% weight loss, and among current weight loss medication users, the prevalence and odds for diet and physical activity behaviors.

**Results:**

10.2% of obese women and 3.1% of obese men reported using prescription weight loss medications in the past 2 years. Of users, 28.2% had lost at least 10% of their pretreatment body weight. The odds of losing at least this much weight were higher among women, those who usually consumed ≥ 5 fruits and vegetables daily and those who met physical activity recommendations. Among current prescription weight loss medication users, 26.7% reported both eating fewer calories and meeting recommended leisure-time physical activity levels (<40% of any group met both). Of those meeting both recommendations, almost half (47.2%) had lost 10% of their pretreatment body weight. Of current users, 9% reported using the medications for weight maintenance.

**Conclusions:**

Only 26.7% of prescription weight loss medication users reported following recommended diet and physical activity behaviors. Further research is needed to assess whether behavioral changes are associated with greater weight loss and maintenance among prescription weight loss medication users.

## Background

Lifestyle modifications, including behavior therapy, diet, and physical activity, are the cornerstone of weight management [1]. Current weight management guidelines state that prescription weight loss medication only may be used as part of a comprehensive weight loss program including diet and physical activity modifications [1]. These modifications include increasing physical activity to 30 to 45 minutes on most days of the week and reducing daily caloric intake by 500 to 1000 calories. Clinical research also suggests that increasing consumption of whole grains, fruits, and vegetables in place of calorie-dense foods may increase satiety and decrease overall caloric intake aiding weight management [2].

The initial goal of weight loss therapy is to reduce body weight by approximately 10% over a 6-month period [1]. Even small losses in weight, such as 5% to 10%, have been found to reduce blood pressure, cholesterol and triglycerides levels, and reduce blood glucose levels among overweight and obese persons without diabetes [3,4]. Weight loss attributable to prescription weight loss drugs is modest, about 3% to 8% of body weight, and is based on a small number of patients assigned to drug treatment in clinical trials [5-8]. Lifestyle modification in combination with pharmacotherapy has been shown to improve weight loss more effectively than pharmacotherapy alone [9]. However, in the general population, data suggests that only one-fifth of individuals who were trying to lose weight reported using the recommended combination of eating fewer calories and meeting weekly physical activity recommendations [10].

Using population-based data we estimated that 4.6 million American adults used prescription weight loss medications between 1996 and 1998 [11]. During this period, obesity medications included phentermine, fenfluramine, dexfenfluramine, and sibutramine. Because of the paucity of population-based data on behaviors among persons using prescription weight loss medications, the purpose of our study was to further examine demographic and behavioral characteristics related to the reported use of prescription weight loss medications collected in the late 1990s as part of a national dataset. These data were collected the same year as issuance of guidelines for assessment and treatment of obesity and therefore provide baseline estimates. Specifically, we examined 1) prescription weight loss medication use by demographic and behavioral characteristics, 2) 10% weight loss among prescription weight loss medication users by demographic and behavioral characteristics, and 3) diet and physical activity behaviors among those who used prescription weight loss medications at the time of the survey.

## Methods

We examined data from the 1998 Behavioral Risk Factor Surveillance System (BRFSS). The BRFSS is a telephone survey of health practices that is conducted each year by all state health departments. Each state, the District of Columbia, and Puerto Rico select an independent probability sample of noninstitutionalized residents aged 18 years and older. In 1998, 149,806 persons responded to the BRFSS; several investigators have published detailed descriptions of survey methods and quality control indices [12]. The average 1998 state cooperation rate (completed interviews divided by completed, refused, and terminated interviews) was 73.4% (range, 45.4%–95.4%).

To determine prescription weight loss medication use, respondents were asked, "In the past two years, have you taken any weight loss pills prescribed by a doctor? Do not include water pills or thyroid medications." Responses were coded as: 1) "Yes, I am currently taking them", 2) "Yes, I have taken them but I am not currently taking them", or 3) "No, I have not taken them".

Respondents with a positive response were asked about their prepill weight, "How much did you weigh just before you started taking prescription weight loss pills for the first time?"

At the end of the interview, respondents were asked to report their current height and weight without shoes. We used current weight and pretreatment weight to calculate current and pretreatment body mass index (BMI), respectively. BMI was calculated as weight (either current or pretreatment) in kilograms divided by current height in meters squared. BMI was categorized into 3 groups: normal weight (<25.0), overweight (25.0–29.9), and obese (≥ 30.0) [1]. Percent weight loss was defined as the difference between pretreatment body weight and current body weight [pretreatment body weight minus current body weight divided by pretreatment body weight times 100]. A 10% weight loss was used in the analyses as this is considered a reasonable individual initial weight loss goal by current clinical guidelines [1]. We used a 6-item question screener to assess the usual consumption of five or more fruits and vegetables per day [13]. Respondent were asked, 1) "How often do you drink fruit juices such as orange, grapefruit, or tomato? 2) Not counting juice, how often do you eat fruit? 3) How often do you eat green salad? 4) How often do you eat potatoes not including French fries, fried potatoes, or potato chips? 5) How often do you eat carrots?", And 6) "Not counting carrots, potatoes, or salad, how many servings of vegetables do you usually eat?"

We used the following questions to determine weight control strategy, "Are you now trying to lose weight?" Those who responded "no" were then asked, "Are you now trying to maintain your current weight, that is to keep from gaining weight?" Additional questions on dietary practices and physical activity were asked of the subset of respondents (95,100) who responded "yes" to trying to lose or maintain their body weight. These respondents were asked "Are you eating either fewer calories or less fat to lose/maintain weight?" Their responses were either: 1) "Yes, fewer calories", 2) "Yes, less fat", 3) "Yes, fewer calories and less fat", or 4) "No". They were also asked "Are you using physical activity or exercise to lose/maintain weight? Their responses were either "yes" or "no".

All individuals were also asked "In the past 12 months, has a doctor, nurse, or other health professional given you advice about your weight?" Responses were "Yes, lose weight", "Yes, gain weight", "Yes, maintain current weight", "No". In an earlier section individuals were asked, "About how long has it been since you last visited a doctor for a routine checkup? The latter question was used to distinguish those who had not received advice due to not seeing a doctor for a routine checkup in the past 12 months.

For respondents who were currently using prescription weight loss medication and were trying to lose weight or maintain their current weight, we attempted to determine if they were meeting the minimal lifestyle recommendations. Respondents who stated they were eating fewer calories – either as fewer calories or as both fewer calories and less fat – were classified as meeting minimal dietary recommendations. Respondents were asked about the two physical activities or exercises they engage in most often and about the frequency, duration, and distance (as appropriate) of each activity [14]. Responses were then classified as one of 56 selected activities. Moderate activity was defined as any of the 56 selected activities, and vigorous activity was defined as aerobic physical activity classified as vigorous-intensity based on estimated metabolic expenditure (MET). To classify an activity as vigorous, it must be aerobic with an assigned MET value that was at least 60% of a person's maximal cardiorespiratory capacity. To have achieved recommended levels of physical activity, a person must have reported engaging in moderate-intensity physical activity ≥ 5 times per week for ≥ 30 minutes each time, vigorous-intensity physical activity ≥ 3 times per week for ≥ 20 minutes each time, or both during the preceding month. Persons reporting some activity during the preceding month but not enough to be classified as moderate or vigorous were classified as insufficient. Persons classified as inactive reported no physical activity outside of their occupation during the preceding month. In addition to these activity categories, we calculated minutes per week of leisure-time (non-occupational) physical activity levels.

We also requested demographic and socioeconomic information such as age in years, sex, race/ethnicity, marital status, educational level, and household income. Of the 149,806 respondents, we excluded those who did not report information on prescription weight loss medication use (n = 1561), had a missing current weight (n = 5229) or height (n = 1369), were pregnant (n = 1780), were missing current weight goal (trying to lose weight or trying to maintain weight) (n = 749), or were missing information on sociodemographic variables, fruit and vegetable intake, or physical activity (n = 3595). We excluded an additional 88 respondents that had a questionable reported weight, height or BMI that was outside the sex-specific reference values from the Third National Health and Nutrition Examination Survey, 1989–1994 [15]. The final study sample included a total of 135,435 respondents.

We used SAS and SUDAAN for the statistical analysis to account for the complex sampling design [16,17]. Student's t tests were used to test between-group differences for means and chi-square tests were used to test between-group differences for proportions. We set statistical significance at P < 0.05 for all comparisons. Multivariate logistic regression analyses were carried out to examine independent predictors of our outcome measures including pharmacotherapy use, 10% weight loss, and meeting diet and physical activity recommendations. Models of pharmacotherapy use were stratified by sex and adjusted for age, race/ethnicity, BMI, education level, household income, marital status, and geographic region. Models of 10% weight loss adjusted for sex, age, race/ethnicity, BMI, education level, household income, marital status, geographic region, smoking status, usual fruit and vegetable consumption, and past month leisure-time physical activity. Models of meeting both diet and physical activity recommendations adjusted for sex, age, race/ethnicity, BMI, education level, household income, marital status, geographic region, and smoking status.

## Results

The results of our multivariate analyses show that the reported use of prescription weight loss medications in the past 2 years was higher among women (4.0%) than men (0.9%). For women, the odds of use was 19% lower among those aged 35–54 years and 75% lower among those 55 years old than among those aged <35 years. The odds of prescription weight loss medication use was 34% lower among non-Hispanic black women than among non-Hispanic white women; and 53% and 39% lower among those women residing in the Northeast and Midwest regions of the United States, respectively, than among women residing in the West region (Table 1). We also found the odds of prescription weight loss medication use was 62% higher among women with some college education than among those with less than a high school education. For men, the odds of use was 62% higher in Hispanics than among non-Hispanic whites.

**Table 1:** Prevalence of Prescription Weight Loss Medication Use in the Previous 2 Years by Characteristics.

	Women			Men		
	(N = 78127)			(N = 57308)		
	n	% (SE)	OR* (95% CI)	n	% (SE)	OR* (95% CI)
Characteristic						
Age group (yrs)						
≥ 55	26787	1.61 (0.14)	0.25 (0.20–0.32)	16484	0.87 (0.13)	0.92 (0.58–1.47)
35–54	31256	5.54 (0.22)	0.81 (0.71–0.93)	24320	1.06 (0.09)	1.03 (0.73–1.45)
18–34	20084	4.80 (0.23)	1.00	16504	0.64 (0.10)	1.00
						
Race/ethnicity						
Other	2613	2.80 (0.72)	0.69 (0.40–1.13)	2245	0.26 (0.10)	0.41 (0.20–0.86)
Hispanic	6248	5.31 (0.48)	1.12 (0.91–1.38)	4447	1.26 (0.27)	1.62 (1.02–2.57)
Non-Hispanic black	7086	3.90 (0.35)	0.66 (0.54–0.80)	4027	0.58 (0.15)	0.69 (0.40–1.19)
Non-Hispanic white	62180	3.93 (0.12)	1.00	46589	0.88 (0.07)	1.00
						
Body mass index (kg/m2)						
≥ 30	14296	10.2 (0.42)	2.27 (1.98–2.60)	10566	3.11 (0.26)	6.24 (4.43–8.79)
25.0–29.9	22005	4.88 (0.23)	1.00	26036	0.51 (0.08)	1.00
<25.0	41826	1.45 (0.10)	0.24 (0.20–0.28)	20706	0.20 (0.04)	0.41 (0.24–0.69)
						
Education level						
College graduate	19203	3.50 (0.23)	1.13 (0.86–1.48)	17525	0.92 (0.11)	1.00 (0.61–1.64)
Some college	22179	5.24 (0.25)	1.62 (1.28–2.06)	15151	0.93 (0.13)	1.04 (0.63–1.69)
High school	26614	3.83 (0.18)	1.26 (1.00–1.59)	17840	0.75 (0.10)	0.85 (0.52–1.39)
<High school	10026	3.00 (0.30)	1.00	6734	0.92 (0.19)	1.00
						
Household income						
Don't know/refused	11947	2.14 (0.21)	0.88 (0.69–1.13)	6402	0.55 (0.18)	0.90 (0.42–1.95)
≥ $75,000	6859	5.13 (0.41)	2.06 (1.60–2.65)	7523	1.41 (0.19)	2.24 (1.38–3.62)
$50–74,999	9044	5.24 (0.38)	1.68 (1.34–2.10)	8690	1.03 (0.15)	1.51 (0.91–2.52)
$35–49,999	12108	5.19 (0.35)	1.54 (1.24–1.92)	10975	0.88 (0.16)	1.28 (0.75–2.19)
$20–34,999	19766	3.91 (0.22)	1.16 (0.97–1.39)	14929	0.68 (0.10)	0.99 (0.61–1.62)
<$20,000	18403	3.52 (0.24)	1.00	8789	0.72 (0.16)	1.00
						
Marital status†						
Married	39608	4.28 (0.16)	0.90 (0.79–1.04)	33318	1.00 (0.08)	1.02 (0.74–1.42)
Not married	38428	3.72 (0.1)	1.00	23923	0.65 (0.09)	1.00
						
Geographic region						
South	17887	5.11 (0.34)	0.90 (0.77–1.07)	14385	0.86 (0.17)	1.3 (0.83–2.04)
Northeast	26747	4.70 (0.18)	0.47 (0.37–0.60)	18007	1.11 (0.11)	0.85 (0.50–1.44)
Midwest	13508	2.32 (0.22)	0.61 (0.51–0.73)	10165	0.72 (0.12)	0.71 (0.44–1.15)
West	18524	3.48 (0.21)	1.00	13839	0.62 (0.08)	1.00

*Models are adjusted for age, race/ethnicity, body mass index, education level, household income, marital status, and geographic region.

^†^Marital status: respondents were classified as "not married if they were divorced, widowed, separated, never been married, member of an unmarried couple.

Women who were obese had more than two times the odds of prescription weight loss medication use compared to women who were overweight and men who were obese had more than six times the odds of use compared to men who were overweight. In addition, women and men who had an annual household income of at least $75,000 had more than two times the odds of prescription weight loss medication use compared to those respondents with an annual household income <$20,000.

At the time of the survey, the average percentage of body weight that was reported lost by prescription weight loss medication users was 8.1% for women and 7.0% for men. About one fifth of users gained weight (women: 19.8%, men: 16.5%), one tenth lost no weight (women: 12.9%, men: 13.4%), one-tenth lost between 1% and 5% (women: 12.7%, men 21.0%), and one fifth lost between 5% and 9% of body weight (women: 19.6%, men: 24.1%), data not shown.

Additionally among users, one-third of women (35.0%) and one quarter of men (25.0%) reported a 10% weight loss, Table 2. The odds of having a 10% weight loss was 37% less among men than women and 43% less among those 55 years old and older than among those 18–34 years. Those who were obese at pretreatment had more than twice the odds of having a 10% weight loss compared to those who were overweight. The odds of having a 10% weight loss was 39% higher among those who were current smokers compared to non-smokers. Those who met minimal recommendations for leisure-time physical activity had more than two twice the odds compared to those who were inactive. The odds of a 10% weight loss was 64% higher among those who usually consumed 5 or more fruits and vegetables daily than among those who usually consumed less than 3 fruits and vegetables a day. Inclusion of weekly minutes of physical activity instead of the meeting physical activity recommendations variable into the 10% weight loss final model resulted in significant odds ratios for individuals with 150–<320 minutes odds ratio (OR) 1.50 (95% confidence interval (C.I.) 1.09–2.17), 320–<420 minutes OR 2.10 (95% C.I. 1.33–3.31), and OR 1.61 (95% C.I. 0.98–2.64) compared to those individuals reporting less than 150 minutes.

**Table 2:** Prevalence of 10% Weight Loss Among Past 2 Year Users of Prescription Weight Loss Medication.

Characteristic	n	% (SE)	OR* 95% CI
Sex			
Men	497	25.0 (2.8)	0.63 (0.44–0.92)
Women	3012	35.0 (1.4)	1.00
			
Age group (yrs)			
≥ 55	506	23.1 (2.9)	0.57 (0.38–0.85)
35–54	1927	34.0 (1.8)	0.95 (0.73–1.24)
18–34	1076	36.7 (2.3)	1.00
			
Race/ethnicity			
Hispanic/other	350	30.4 (7.4)	0.77 (0.53–1.14)
Non-Hispanic black	258	35.2 (4.6)	0.76 (0.50–1.15)
Non-Hispanic white	2404	35.6 (1.6)	1.00
			
Pretreatment body mass index (kg/m2)			
≥ 30	1799	45.5 (2.0)	2.24 (1.17–2.94)
25.0–29.9	269	27.2 (2.3)	1.00
<25.0	292	7.1 (1.7)	0.22 (0.13–0.38)
			
Education level			
College graduate	675	33.1 (2.9)	1.00 (0.62–1.61)
Some college/tech	1106	37.2 (2.5)	1.16 (0.74–1.82)
High school	993	35.9 (2.4)	1.35 (0.89–2.06)
<High school	237	27.5 (4.4)	1.00
			
Household income			
Don't know/refused	231	32.8 (5.1)	0.80 (0.46–1.37)
≥ $75,000	343	33.0 (3.9)	0.66 (0.41–1.06)
$50–74,999	467	33.4 (3.4)	0.65 (0.41–1.01)
$35–49,999	625	32.2 (3.1)	0.66 (0.44–1.00)
$20–34,999	800	36.5 (2.9)	0.82 (0.57–1.19)
<$20,000	564	39.9 (3.6)	1.00
			
Marital status			
Married	1696	33.4 (1.8)	0.92 (0.70–1.21)
Not married	1314	37.4 (2.4)	1.00
				
Geographic region			
South	1184	32.9 (1.8)	1.01 (0.74–1.39)
Northeast	243	30.9 (4.8)	0.91 (0.70–1.21)
Midwest	653	40.9 (3.2)	1.13 (0.79–1.64)
West	884	34.9 (3.3)	1.00
			
Smoking status			
Current smoker	695	39.8 (3.2)	1.39 (1.04–1.86)
Former smoker	709	30.1 (2.8)	0.87 (0.65–1.17)
Never smoker	1608	34.7 (1.9)	1.00
			
Usual fruit and vegetable consumption			
≥ 5 day	693	29.1 (3.1)	1.64 (1.19–2.27)
3–4 day	1205	35.4 (2.3)	1.16 (0.90–1.51)
<3 day	1114	30.2 (4.4)	1.00
			
Past month leisure-time physical activity†			
Meeting recommendations	1331	42.9 (2.2)	2.19 (1.60–2.98)
Insufficient	834	30.3 (2.5)	1.16 (0.83–1.60)
Inactive	847	26.6 (2.7)	1.00

*Models are adjusted for sex, age, race/ethnicity, body mass index, education level, household income, marital status, geographic region, smoking status, usual fruit and vegetable consumption, and leisure-time physical activity in the past month.

^†^To have achieved recommended levels of physical activity, a person must have reported engaging in moderate-intensity physical activity ≥ 5 times per week for ≥ 30 minutes each time, vigorous-intensity physical activity ≥ 3 times per week for ≥ 20 minutes each time, or both during the preceding month [14].

We also assessed professional advice, diet, and physical activity among current prescription weight loss medication users by current weight intention (Table 3). Although most of the respondents who reported current medication use were trying to lose weight, 9% reported they were trying to maintain weight. Examination of past year health care professional advice about weight among those who had seen a doctor for a routine checkup showed that almost three quarters of those trying to lose weight had been advised to lose weight. However, almost one-quarter of current prescription weight loss medication users who were trying to lose weight were given no advice about their weight. Over half of users who were trying to maintain their weight were given no advice about their weight. Assessment of usual fruit and vegetable consumption showed that about one quarter of those trying to lose weight (28.3%) and one quarter of those trying to maintain their weight (27.6%) reported consuming 5 fruits and vegetables a day. Almost two thirds of current prescription weight loss medication users who reported they were trying to lose weight reported reducing caloric consumption (62.0%), 70.9% reported increasing physical activity, and 47.3% met physical activity recommendations. However, these behaviors were reported less often in current prescription weight loss medication users who reported they were trying to maintain their weight, 39.6%, 49.6%, and 29.9%, respectively. A similar proportion of weekly minute categories were observed among those trying to lose weight and those trying to maintain weight, with 8.0% and 9.7% respectively, reporting at least 420 minutes per week.

**Table 3:** Prevalence of Professional Advice, Diet, and Physical Activity Behaviors Among Current Users of Prescription Weight Loss Medication.

	Current Weight Management Intention		
	Lose Weight	Maintain Weight	Total
	(n = 563)	(n = 59)	(n = 622)
	% (SE)	% (SE)	% (SE)

Health professional past year weight advice*			
Lose weight	73.9 (3.6)	35.6 (10.7)	71.0 (3.4)
Maintain weight	1.9 (0.6)	7.2 (5.2)	2.5 (0.7)
Gain weight	0.1 (0.1)	0.0 (0.0)	0.3 (0.2)
No advice	24.1 (3.5)	57.2 (10.6)	26.3 (3.3)
			
Diet			
Fewer calories for weight control	62.0 (3.2)	39.6 (7.5)	60.2 (3.0)
≥ 5 fruits and vegetables daily	28.3 (3.4)	27.6 (7.7)	28.2 (3.1)
			
Physical activity			
Increased physical activity			
for weight control	70.9 (3.4)	49.6 (9.3)	69.1 (3.2)
Past month			
Inactive	25.1 (3.5)	47.9 (9.5)	27.0 (3.3)
Insufficient	27.6 (3.3)	22.2 (7.1)	27.2 (3.1)
Meeting recommendations**	47.3 (3.8)	29.9 (7.7)	45.8 (3.5)
			
Past month (weekly minutes)			
<150 minutes	53.0 (4.1)	58.4 (11.1)	52.1 (3.9
150–<320 minutes	27.9 (3.8)	20.2 (8.0)	27.5 (3.5)
320–<420 minutes	11.1 (2.8)	11.7 (5.6)	11.1 (2.6)
≥ 420 minutes	8.0 (1.9)	9.7 (7.1)	9.4 (1.9)

*Among those who reported that had visited a doctor for a routine checkup in the past 12 months (n = 514).

*†To have achieved recommended levels of physical activity, a person must have reported engaging in moderate-intensity physical activity ≥ 5 times per week for ≥ 30 minutes each time, vigorous-intensity physical activity ≥ 3 times per week for ≥ 20 minutes each time, or both during the preceding month [14].

Only one-quarter (26.7%) of current prescription weight loss medication users met both lifestyle recommendations – eating fewer calories and attaining recommended physical activity levels. Our results indicate that among current users, the odds of meeting both recommendations was 62% lower among men than among women and 56% lower among other race/ethnic groups than among non-Hispanic whites (Table 4). We also found that the odds of meeting both recommendations was more than three time higher among those with a household income of at least $75,000 (OR 3.23) compared to those with a household income <$20,000 and those who usually consumed 5 or more fruits and vegetables a day had twice the odds compared to < 3 fruits and vegetables a day. In a sensitivity analysis, we found that adding to this model past year health professional advice about weight among those who had a routine checkup (n = 559) did not appreciably change the results and that advice to lose weight was not an independent predictor of meeting both recommendations (OR = 1.37, 95% C.I. 0.73–2.56).

**Table 4:** Meeting diet (fewer calories) and Physical Activity Recommendations* Among Current Users of Prescription Weight Loss Medication.

Characteristic	n	Total (SE)	OR† 95% CI
Sex			
Men	98	14.8 (4.4)	0.38 (0.17–0.84)
Women	524	29.7 (3.3)	1.00
Age group (yr)			
≥ 55	91	21.6 (6.9)	0.92 (0.34–2.46)
35–54	338	29.5 (4.1)	1.10 (0.58–2.11)
18–34	193	25.7 (4.1)	1.00
Race/ethnicity			
Other	154	16.9 (4.8)	0.44 (0.20–0.96)
Non-Hispanic white	468	32.1 (3.4)	1.00
Pretreatment body mass index (kg/m2)			
≥ 30.0	403	25.7 (3.1)	1.02 (0.54–1.91)
<30.0	187	28.8 (6.0)	1.00
Education level			
College graduate	147	29.8 (6.4)	1.78 (0.76–4.15)
Some college/tech	201	34.7 (5.1)	1.97 (1.00–3.90)
<High school	274	19.7 (4.0)	1.00
Household income			
≥ $75,000	91	39.0 (7.4)	3.23 (1.18–8.83)
$50–74,999	80	29.0 (2.9)	2.49 (0.85–7.25)
$35–49,999	140	32.4 (5.9)	2.45 (0.82–7.35)
$20–34,999	157	27.1 (4.7)	2.14 (0.89–5.14)
<$20,000‡	154	14.9 (3.9)	1.00
Marital status			
Married	370	28.0 (4.9)	0.87 (0.47–1.59)
Not married	252	24.6 (4.0)	1.00
Geographic region			
South	241	27.6 (4.0)	0.78 (0.34–1.78)
Midwest/Northeast	189	26.1 (4.3)	1.03 (0.45–2.32)
West	159	26.5 (6.3)	1.00
Usual fruit and vegetable consumption			
≥ 5 day	158	34.7 (6.4)	2.30 (1.05–5.03)
3–4 day	227	26.3 (4.1)	1.30 (0.67–2.53)
<3 day	237	21.3 (3.8)	1.00
Smoking			
Current smoker	149	31.5 (6.6)	1.78 (0.94–3.36)
Former smoker	146	26.4 (5.5)	1.10 (0.54–2.26)
Never smoker	327	24.3 (3.3)	1.00

*To have achieved recommended levels of physical activity, a person must have reported engaging in moderate-intensity physical activity ≥ 5 times per week for ≥ 30 minutes each time, vigorous-intensity physical activity ≥ 3 times per week for ≥ 20 minutes each time, or both during the preceding month [14].

^†^Models are adjusted for sex, age, race/ethnicity, body mass index, education level, household income, marital status, geographic region, smoking status, and usual fruit and vegetable consumption.

^‡^Includes "Don't know" and "Refused" annual household income responses.

In a secondary analyses we assessed prevalence of a 10% weight loss among current prescription weight loss medication users who were trying to lose weight (not maintain) by whether or not recommendations were met for eating fewer calories and physical activity (n = 237). 39.6% (SE 5.3) of individuals who reported both eating fewer calories and who met physical activity recommendations had lost at least 10% of their pretreatment weight; 36.5% (SE 6.4) who reported eating fewer calories but who did not meet the physical activity recommendations had lost at least 10% of their pretreatment weight; 54.3% (SE 9.8) who reported not eating fewer calories but met physical activity recommendations had lost at least 10% of their pretreatment weight; and 25.5% (SE 5.9) who did not meet either lifestyle recommendation had lost at least 10% of their pretreatment weight, (chi-square p = 0.03).

## Discussion

Although in 1998 clinical guidelines recommended that prescription medication for obesity be used in conjunction with lifestyle modifications [1], we found that during that same year only one quarter of current prescription weight loss medication users reported minimal diet (eating fewer calories) and physical activity behaviors recommended for weight loss. Our analyses indicated that meeting these recommendations was low across all sociodemographic groups (<40%) and, men were less likely to meet recommendations compared to women.

Although patterns of prescription medication use differed by sex, for both men and women the odds of use was higher among those who were obese compared to those who were overweight although at different magnitudes. Our data show than more overweight women used prescription weight loss medications (4.88%) than did obese men (3.11%). It is possible that pharmacotherapy prescribing patterns differ by sex since more women than men attempt weight loss [10] or that for men, a higher BMI is needed before personal action regarding weight is taken.

Eating fruits and vegetables and taking part in recommended levels of physical activity through leisure-time activities were more frequent behaviors among prescription weight loss medications users who had lost at least 10% of their body weight. Although we had no direct measurements of caloric intake, fruits and vegetables in their natural state are low in energy density. Thus, adding or substituting fruits and vegetables for energy-dense snacks and sweets can impact satiety and weight gain [2,18,19]. Our analyses also indicate that those respondents who met the minimal recommendations for leisure-time physical activity had twice the odds of having lost 10% of their body weight compared to those who were sedentary. We observed higher odds of 10% weight loss for those with at least 150 weekly minutes, but the magnitude did not appear to have a dose effect. We believe additional research is needed to determine whether physical activity modifications require greater duration, such as exercising 60 minutes or more daily to prevent weight regain as has been suggested by the Institute of Medicine [20], to improve the efficacy of currently approved obesity pharmacotherapy for weight management.

About 40% of current prescription weight loss medications users who both reduced calories and met leisure-time physical activity recommendations had lost 10% of their body weight. Our data also show that respondents who consumed usually less than 5 fruits and vegetables per day were less likely to meet both diet and physical activity recommendations than did those who consumed 5 or more fruits and vegetables a day. Because fewer than half of physicians counsel obese patients about weight control [21,22] we believe the opportunity exists for physicians and other health care professionals to provide counseling that emphasizes appropriate weight control practices that include regular physical activity and a balanced low-calorie diet which includes increasing consumption of fruits and vegetables.

The current study had several limitations. Although the participants self-reported pre-medication body weight and current weight, we did not ask for the date prescription weight loss medication use began and it could not be established whether weight loss was solely attributable to pharmacotherapy usage. We were also unable to determine if respondents had lost a substantial amount of weight but then regained the weight (weight history). Self-reported weights and weight history may also be a concern because individuals, especially those who are overweight, may underreport their weight [23]. We lacked dietary assessment measures to quantify the number of calories consumed. Thus, we could not determine whether individuals had actually reduced their caloric consumption to levels that would lead to substantial weight loss. Research suggests that some obese subjects underestimate their caloric intake and overestimate their physical activity [24]. In addition, we lacked data on whether respondents were counseled specifically about physical activity and/or nutrition for weight management or had undergone behavioral therapy.

Although we found that about one fifth of participants had lost between 5% and 9% of their pretreatment body weight and one third of participants had lost at least 10% of their pretreatment body weight, the pill dosage and length of medication use were not collected. Thus, some users may have used pills for a short time, such as less than 6 months, and would not be expected to have lost 5% to 10% of their body weight.

In current practice, physicians treating obesity can prescribe phentermine, sibutramine, and orlistat (available April 1999). Analysis of national data on patient visits found that phentermine was the most common antiobesity medication in 2001 and in early 2002 (based on January through March figures) [25], suggesting that our data, although collected in 1998, has relevance to current weight management.

Most nonsurgical obesity treatments lead to weight loss for the first 6 months followed by regain [26]. Thus, some health care professionals suggest that prescription weight loss medication be prescribed either to enhance weight loss during the active weight-loss phase or to prevent later regain [26,27]. In our study, 9% of current medication users reported that they were currently trying to maintain their weight. We observed that the behaviors of these individuals differed from those who said they were currently trying to lose weight, e.g. fewer reported eating less calories, fewer met physical-activity recommendations, and fewer were advised by a health care professional to either lose or maintain weight. Additional research is needed on use of pharmacotherapy for weight maintenance.

## Conclusions

We found that one in ten obese women and one in 33 obese men reported using a prescription weight loss medication in the past two years. Among medication users, one-third of women and one quarter of men reported a 10% weight loss. Only one quarter of current medication users reported the minimal diet and physical activity behaviors recommended for weight loss. Ongoing population-based surveillance is needed to assess the prevalence of prescription weight loss medication use and whether pharmacotherapy users are making appropriate lifestyle changes in order to lose or maintain weight.

## List of abbreviations

BRFSS: Behavioral Risk Factor Surveillance System

BMI: body mass index

OR: odds ratio

95% C.I.: 95% confidence interval

## Competing interests

The author(s) declare that they have no competing interests.

The questions that were asked of all authors:

Have you received reimbursements, fees, funding, or salary from an organization that may in any way gain or lose financially from the publication of this paper in the past five years? No.

Have you held any stocks or shares in an organization that may in any way gain or lose financially from the publication of this paper? No.

Do you have any other financial competing interests? No.

Are there any non-financial competing interests you would like to declare in relation to this paper? No.

## Author contributions

HB performed the statistical analysis and drafted the manuscript.

LKK conceived the study questions, provided statistical input, and edited the manuscript.

MKS conceived the study questions, provided statistical input, and edited the manuscript.

All authors read and approved the final manuscript.
